# Problematic media use is associated with poor oral health in Turkish school-age children: a pilot cross-sectional study

**DOI:** 10.1186/s12903-023-03238-x

**Published:** 2023-07-28

**Authors:** Şeyma Mustuloğlu, Özlem Tezol

**Affiliations:** 1 Department of Pediatric Dentistry, Faculty of Dentistry, Mersin University, Mersin, Turkey; 2 Department of Pediatrics, Faculty of Medicine, Mersin University, Mersin, Turkey

**Keywords:** Children, Media use, Oral habits, Oral health

## Abstract

**Objectives:**

In the recent years, problematic media use (PMU) has become a serious health concern for children. The precisely defined effects of PMU on children’s oral health are unknown. It was aimed to investigate the relationship between the PMU and oral health and oral habits in school-age children.

**Methods:**

In this cross-sectional study, mothers and their healthy children aged 6–11 years who applied to pediatric dentistry outpatient clinic were enrolled. PMU was assessed using the parent-reported Problematic Media Use Measure Short Form (PMUM-SF). PMUM-SF scores were divided into 3 groups from the lowest to the highest tertile. Multivariable logistic regressions for PMU (moderate-high vs. low) were used to predict the odds of having good and parafunctional oral habits, poor oral hygiene, gingivitis and caries.

**Results:**

Totally 153 mother–child pairs participated in this study. Plaque index, gingival index, ICDAS-II (International Caries Detection and Evaluation System), DMFT and DMFS [decayed (D), missing (M), filled (F) tooth (T) /surfaces (S)] scores were significantly higher in children with moderate-high PMU (*P* < 0.05). After adjusting for potential confounders, moderate-high PMU decreased the probability of good oral habit of daily toothbrushing [Odds (95% CI) = 0.43 (0.20–0.94)] while it increased the risk of oral parafunctional habit of object sucking/biting [Odds (95% CI) = 3.34 (1.27–8.74)]. Moderate-high PMU increased the risk of moderate-severe gingivitis, moderate-extensive caries and the presence of DMFT [Odds (95% CI) = 2.13 (1.01–4.50); 4.54 (1.11–18.54) and 2.16 (1.07–4.36), respectively].

**Conclusions:**

Turkish school-age children with a remarkable PMU were significantly more likely to have poor oral health and exhibit oral parafunctional habits Oral health screening seems to be needed for Turkish children experiencing PMU.

## Introduction

Dental caries, which affects 60–90% of children worldwide, is recognized as one of the most common chronic disorders among children [1]. It is, also, the primary reason for dental pain and tooth loss [2]. In addition, if dental caries is not treated, it can negatively affect children's ability to chew, food choice, communication, school participation, concentration, and reduce their quality of life. In addition to these, it is possible to control the disease in its early stages by using fluoride, proper eating habits, and removing plaque on the tooth surface with regular brushing [2, 3]. The American Dental Association (ADA) recommends brushing twice a day for 2 min with a soft-bristled toothbrush and fluoride toothpaste, limiting sugary drinks and snacks and taking regular dental checkups to maintain oral health [4].

Most of the risk factors for chronic disease such as dental caries arise in childhood and are usually lifestyle related. Adopting healthy behaviors at an early age is an important step in maintaining healthy habits throughout life [5]. Since the development of oral hygiene habits in childhood can be affected by other behaviors such as eating, sleeping, and watching/ using screens of multimedia devices [6], discussing the possible effects of these behaviors on oral health may contribute to the improvement of public oral health.

At the present time, the younger generation is utilizing electronic media as an important part of their lives [7]. Besides the advantages of digital media devices in terms of accessing information and fast communication, they are often used for entertainment among children and adolescents. In addition, the increase in the variety of these devices and the fact that they are easily accessible from anywhere at any time cause children to use their screen increasingly [8]. When the increased use of screen media reaches an “addictive level”, the concept of problematic use of screen media develops and it leads to concerns in their parents, family members, children and adolescents themselves [9]. Problematic media use (PMU) is a form of dependence on media use for children aged 12 years and below which distinguishes excessive media use that interferes with a child's functioning from benign media use and it has become a serious health concern for children [10]. PMU in children can cause many social, psychological, and health problems such as conflict with parents and siblings about media use, delaying or avoiding schoolwork, disruption in the in-person peer interaction, obesity, sleep disturbance, and physical inactivity [11–13]. The measure of PMU reveals the overall functioning of the child above total daily screen time and type of media used by measuring elements of addictive media use among children including preoccupation, withdrawal and unsuccessful attempts of the parents to control their child [11].

Screen time has been reported to be associated with dental caries in children [14]. Also, poor oral health was found to be associated with early exposure to screens and long-term screen use in Turkish preschool children [15]. However, to our knowledge, there is no published research in the literature investigating the probable relationship between PMU and oral health in children. In a comprehensive literature review, it can be seen that PMU has been evaluated most commonly in the context of its effects on sleep, the cardiovascular system, orthopedics, vision as well as psychoneurological and social outcomes among children and adolescents [16]. Since behavioral addictions result in neglect of some aspects of self-care such as personal hygiene [17, 18], we have hypothesized that PMU may also lead to neglect of dental hygiene and poor oral health. Thus, the present study aimed to investigate the relationship of the PMU with oral health and oral habits in school-age children.

## Methods

### Study design and setting

We conducted a descriptive cross-sectional study between 1-April-2022 and 15-June-2022 at the Pediatric Dentistry Department of the Dentistry Faculty, Mersin University. The signed consent forms from the mothers and their children were obtained before their participation. The study procedures were performed in accordance with the Declaration of Helsinki and the local ethics committee of the university approved the study.

### Participants and data collection

**Fig. 1:**
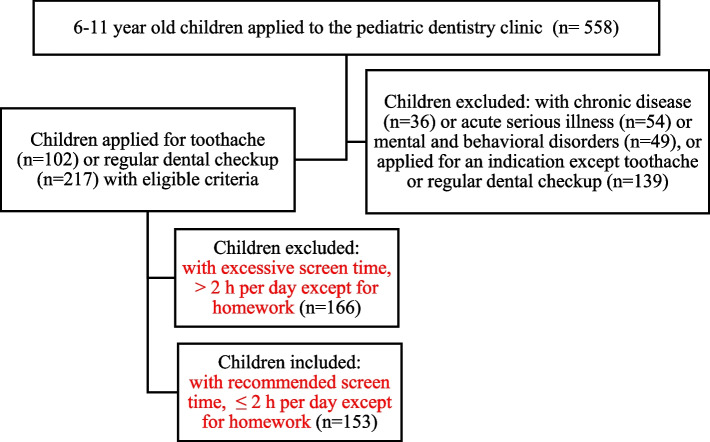
Flowchart of the study population selection

The children aged 6–11 years who were admitted to the pediatric dentistry clinic for toothaches or regular dental checkups and their literate mothers were enrolled in this study. The participation status was determined by asking the mothers about the daily media use of their children in the waiting room. If the mother stated that her child was using media device(s) every day with a media-use time of less than or equal to 2-h per day except spent for homework in the recent month, the study process was described and the mothers were requested to complete a structured survey and the Problematic Media Use Measure Short Form (PMUM-SF). The children who were diagnosed with an acute serious disease, chronic disease or a neuropsychiatric disorder were excluded from the study. Besides, the children reported with a screen time of > 2 h per day except spent for homework were excluded from this study to eliminate the influence of excessive screen time (Fig. 1) [19].

The structured survey form was designed to collect sociodemographic, anthropometric, sleep duration, screen-based media device use and oral care data as general descriptive characteristics. Parental education levels, family income level and settlement type were categorized. Anthropometric z-scores were calculated using the child growth standards of the World Health Organization (WHO) [20]. Ownership of children’s toothbrush, their daily toothbrushing habits, frequency of toothbrushing, type of used toothpaste and frequency of visiting a dentist were questioned. A regular dental checkup was accepted as visiting a dentist once or twice per year. Daily toothbrushing and regular dental checkups were considered as good oral habits. Information related to children’s oral parafunctional habits including thumb sucking, lip sucking/biting, gnashing teeth, biting nails, and object sucking/biting was identified as either present or not present.

### Oral and dental examination

Oral and dental examinations were performed by the first author, who is experienced in pediatric dentistry for 5 years in the dental clinic, under the reflector light as per the guidelines of the WHO. The cavitated carious lesions were evaluated according to decayed (D, d), missing (M, m), filled (F, f) tooth (t)/surfaces (s) indices (dmft/s: for primary dentition and DMFT/S: for permanent dentition) [21]. Furthermore, ICDAS-II (International Caries Detection and Assessment System) was used for a more detailed evaluation of dental caries including early enamel caries lesions and stages of lesions [22]. The maximum score assessed in the mouth was recorded as the ICDAS-max score of the patient (total scores 0; wellness and 1–2, 3–4 and 5–6; initial, moderate, and extensive caries, respectively) [23]. Oral hygiene status of the patients according to plaque index of Silness and Löe (scores 0–3; no plaque, thin, moderate, and heavy plaque, and total scores 0, 0.1–0.9, 1.0–1.9 and 2.0–3.0; excellent, good, fair and poor oral hygiene, respectively), and gingival health status according to gingival index of Silness and Löe (scores 0–3; no, mild, moderate and severe inflammation and total scores 0, 0.1 1.0, 1.1–2.0 and 2.1–3.0; free of mild, moderate, and severe gingivitis, respectively) were assessed [24].

Before the oral and dental examinations, a calibration study was performed by an experienced and trained specialist dentist, and inter-examiner Kappa coefficients were found to be 0.81, 0.91, 0.82 and 0.88 for the PI; GI; ICDAS-II; and DMF index system, respectively. In addition, intra-examiner kappa values for all indices regarding oral health were found to be higher than 0.80.

### The measurement of problematic media use

Problematic Media Use Measure (PMUM) is a parent-reported measure of screen media addiction in children aged between 4 to 11 years. PMUM assesses a unidimensional construct of PMU which consists of items created based on the nine criteria for Internet Gaming Disorder in the DSM-5. The PMUM-SF uses nine items corresponding to these criteria. The responses were based on a 5-point Likert scale ranging between never (1) to always (5). The total score (range, 1–5) is generated by adding the scores from the items and dividing them by 9. A higher PMUM-SF score is linked to more PMU [11]. Furuncu and Öztürk performed reliability and validity study of Turkish version of PMUM and PMUM-SF, and revealed that PMUM-SF has good support for its validity to test screen addiction in Turkish children aged between 4 to 11 years [25].

### Outcome measures

The primary patient-centered outcome of the study was problematic media use in children admitted to a pediatric dentistry clinic. The primary clinical outcomes were decayed, missing, filled teeth in permanent and primary teeth, plaque/inflammation indices, and parafunctional oral habits in these children. Toothbrushing habit status/characteristics and regular dental checkup were also investigated as the secondary clinical measures. Media devices used were non-clinical secondary outcomes.

### Sample size calculation and statistical analyses

Since the relationship between PMU and child oral-dental health in Turkish population was not documented, a moderate correlation was assumed and total sample size was determined as 134 children with an effect size of 0.3 and a power of 95% (G*Power 3.1.9.4). All volunteers who accepted to participate in the study were included until the data collection period expired.

Data were analyzed using SPSS 21 statistics software. The Kolmogorov–Smirnov test and histograms were used to test for the distribution normality of study data. Median (IQR, 25th-75th percentile), mean ± standard deviation and percentage values were expressed. Two parametric values were compared using the Student’s t-test while two nonparametric values were compared using the Mann–Whitney U test. The Chi-square test was used for the categorical variables. Since PMUM-SF has no defined cut-off points, PMUM-SF scores were divided into 3 groups from the lowest to the highest tertile. A Spearman’s rank correlation was used to measure the relationship between PMUM-SF scores and oral health parameters. Multivariate logistic regression for PMU (moderate-high vs. low) was used to predict the odds of having good and parafunctional oral habits, fair-poor oral hygiene, moderate-severe gingivitis, moderate-extensive caries, the presence of decayed/filled/missing teeth, and the presence of decayed/filled surfaces. The odds ratios (ORs) were calculated at a CI of 95% with the ENTER method. In the regression analyses, the independent variables included variables with a *p* < 0.250 in univariate statistics. All statistical assessments were two-sided and statistical significance was considered at *P* < 0.05.

## Results

A total of 153 mother–child pairs participated in this study. The overall median age of the children was 8.8 (6.2–11.8) years and 52.3% were males. According to the statements of the mothers, all 153 children had a screen time of 1.5 to 2 h per day except for spent homework. Twelve (7.8%) children, who gained a PMUM-SF score of 1 which indicated absence of PMU, were categorized into the lower tertile group. The numbers of children with a PMUM-SF score in the lower tertile and the moderate-high tertile were 51 and 102, respectively. No significant difference was present between the children with low and moderate-high PMU in terms of age, gender, birth order, breastfeeding, parent and family characteristics, and anthropometric z-scores (*P* > 0.05). The median duration of daily night-sleep time showed no significant difference between the groups [9 (8–10) h vs. 9 (8–10) h, *P* = 0.857]. Having daily toothbrushing habit was significantly more common in the low-PMU group (70.6% vs. 47.1%, *P* = 0.006). Also, using children’s toothpaste was significantly more common in the low-PMU group (74.5% vs. 51.6%, *P* = 0.010). No significant difference was present between the groups in terms of frequency of having regular dental checkups (19.6% vs. 8.8%, *P* = 0.057). The comparison between the groups in terms of sociodemographic, anthropometric, and oral care characteristics was presented in Table 1.

**Table 1:** The comparison between the groups in terms of sociodemographic, anthropometric, and oral care characteristics

	Low problematic media use (*n* = 51)	Moderate-high problematic media use (*n* = 102)	*P—*value
Age, year	8.3 (7.2–9.7)	9.3 (7.9–10.5)	0.077^a^
Gender, male	47.1	54.9	0.360^b^
Birth order			
1^st^	43.1	45.1	0.818^b^
≥ 2^nd^	56.9	54.9	
Breastfeeding at least six months	100.0	96.1	-
Total duration of breastfeeding, month	12 (6–20)	12 (6–21)	0.577^a^
Pacifier use	49.0	40.2	0.299^b^
Duration of pacifier use, month	0 (0–12)	0 (0–12)	0.331^a^
Feeding bootle use	58.8	58.8	1.00^b^
Duration of feeding bottle use, month	9 (0–14)	7 (0–12)	0.850^a^
Maternal age, year	37.1 ± 5.8	37.4 ± 5.7	0.765^c^
Paternal age, year	42.0 ± 5.8	41.0 ± 5.6	0.312^c^
Maternal occupation, working mom	19.6	18.6	0.884^b^
Maternal educational level			
Primary school	39.2	50.0	0.207^b^
High school/college	60.8	50.0	
Paternal educational level			
Primary school	35.3	41.2	0.482^b^
High school/college	64.7	58.8	
Number of children in the family	2 (2–3)	2 (2–3)	0.357^a^
Family structure, nuclear	84.3	80.4	0.554^b^
Family income level			
High	7.8	6.9	0.974^b^
Middle	43.1	43.1	
Low	49.0	50.0	
Settlement, urban	64.7	56.9	0.352^b^
Z-score			
Height for age	-0.32 (-0.79 to 0.55)	0.21 (-0.51 to 0.74)	0.064^a^
Weight for age	-0.21 (-0.78 to 0.34)	-0.13 (-0.72 to 0.55)	0.539^a^
Body mass index	-0.34 (-0.59 to 0.61)	-0.28 (-0.69 to 0.44)	0.501^a^
Having own toothbrush	100.0	100.0	**-**
Having daily toothbrushing habit	70.6	47.1	**0.006**^**b**^
Toothbrushing frequency			
Never	7.8	10.8	**0.028**^**b**^
Once or twice a week	21.6	42.1	
Once a day	31.4	26.5	
Twice a day	39.2	20.6	
Brushing teeth			
On own	85.1	83.5	**-**
On own under parent	14.9	14.3	
Supervision assisted	0.0	2.2	
Using toothpaste	100.0	100.0	-
Adult toothpaste	25.5	48.4	**0.010**^**b**^
Children's toothpaste	74.5	51.6	
Visiting a dentist			
For the first time	21.6	20.6	0.142^b^
When he/she has a toothache	58.8	70.6	
Regularly once or twice per year	19.6	8.8	

^a^comparison of medians, the Mann–Whitney U test

^b^comparison of percentages, the Chi Square test

^c^comparison of means, the Independent–Samples T test. Showing only the percentage of findings

The median PMUM-SF scores in the low and moderate-high PMU groups were detected to be1.2 (1.1–1.4) and 2.4 (1.9–3.0) (*P* < 0.001), respectively. Use of a smartphone, tablet, and computer was significantly more common in the moderate-high PMU group (*P* < 0.05). The frequency of object sucking/biting was significantly higher in the moderate-high PMU group (30.4% vs. 11.8%, *P* = 0.011). The median values of plaque index, gingival index, and max ICDAS score were significantly higher in moderate-high PMU group (1.3 vs. 1.1, *P* = 042; 1.1 vs. 0.8, *P* = 0.012; and 5.5 vs. 5.0, *P* = 0.029, respectively). Also, DMFT and DMFS scores were significantly higher in the moderate-high PMU group (both 1.0 vs. 0, *P* = 0.018 and *P* = 0.013, respectively). The comparison between the groups in terms of media use characteristics, oral parafunctional habits and oral health parameters can be seen in Table 2.

**Table 2:** The comparison between the groups in terms of media use characteristics, parafunctional oral habits, and oral health parameters

	Low problematic media use (*n* = 51)	Moderate-high problematic media use (*n* = 102)	*P*—value
PMUM-SF score	1.2 (1.1–1.4)	2.4 (1.9–3.0)	** < 0.001**^**a**^
Using media devices			
Television	68.6	67.6	0.902^b^
Smart phone	54.9	74.5	**0.014**^**b**^
Tablet	29.4	46.1	**0.048**^**b**^
Computer	11.8	25.5	**0.049**^**b**^
PlayStation	5.9	5.9	1.00^b^
parafunctional oral habits			
Thumb-sucking	0.0	3.9	0.302^b^
Lip sucking or biting	11.8	21.6	0.139^b^
Gnashing teeth	27.5	30.4	0.707^b^
Biting nail	15.7	21.6	0.388^b^
Object sucking or biting	11.8	30.4	**0.011**^**b**^
Having at least one parafunctional oral habit	25 (49.0)	63 (61.8)	0.133^b^
Number of parafunctional oral habits	1 (1–1.5)	1 (1–2)	0.053^a^
1 habit	76.0	54.0	0.057^b^
≥ 2 habits	24.0	46.0	
Plaque index score	1.1 (0.7–1.5)	1.3 (0.8–1.9)	**0.042**^**a**^
Excellent	0.0	0.0	0.095^b^
Good	47.1	33.3	
Fair	43.1	44.1	
Poor	9.8	22.5	
Oral hygiene			
Good	47.1	33.3	0.099^b^
Fair-poor	52.9	66.7	
Gingival index score	0.8 (0.4–1.2)	1.1 (0.6–1.7)	**0.012**^**a**^
Free of gingivitis	0.0	0.0	**0.036**^**b**^
Mild gingivitis	68.6	51.0	
Moderate gingivitis	29.4	36.3	
Severe gingivitis	2.0	12.7	
Gingivitis			
Mild	(68.6)	51.0	**0.038**^**b**^
Moderate-severe	(31.4)	49.0	
ICDAS max score	5 (4.5–6)	5.5 (5–6)	**0.029**^**a**^
Initial lesions	11.8	3.9	0.052
Moderate lesions	13.7	6.9	
Extensive lesions	74.5	89.2	
Caries			
Initial	11.8	3.9	0.085
Moderate-extensive	88.2	96.1	
DMFT	0 (0–1)	1 (0–2)	**0.018**^**a**^
Absence of decayed/filled/missing	66.7	48.0	**0.029**^**b**^
Presence of decayed/filled/missing	33.3	52.0	
DMFS	0 (0–1)	1 (0–3)	**0.013**^**a**^
Absence of decayed/filled	66.7	48.0	**0.029**^**b**^
Presence of decayed/filled	33.3	52.0	
dmft	4 (1–6.5)	4 (2–6)	0.523^a^
dmfs	11 (2–19)	9.5 (3–17)	0.592^a^

*PMUM-SF* Problematic media use measure-short form, *ICDAS* The international caries detection and assessment system, *D/d* decay, *M/m* Missing, *F/f* Filling, *t/T* Teeth, *s/S* Surface. Showing only the percentage of findings

^a^comparison of medians, the Mann–Whitney U test

^b^comparison of percentages, the Chi Square test

There was a weak correlation between PMUM-SF score and the number of oral parafunctional habits (r = 0.17, n = 153, *P* = 0.030). A moderate-high PMU decreased the probability of good oral habit of daily toothbrushing [Odds (95% CI) = 0.43 (0.20–0.94), *P* = 0.033], while it increased the risk of oral parafunctional habit of object sucking/biting [Odds (95% CI) = 3.34 (1.27–8.74), *P* = 0.014]. There was no relationship between moderate-high PMU and having a regular dental checkup, thumb-sucking, lip-sucking/biting, gnashing teeth and biting nails (*P* > 0.05) (Table 3).

**Table 3:** The relationship of moderate-high problematic media use with oral habits and oral health

	*P* value	OR	95% CI for EXP(B)	
			Lower	Upper
Good oral habits				
Daily tooth brushing	**0.033**	**0.43**	**0.20**	**0.94**
Regular dental checkup	0.062	0.37	0.13	1.05
Parafunctional oral habits				
Lip sucking or biting	0.144	2.10	0.78	5.67
Gnashing teeth	0.703	1.16	0.54	2.51
Biting nail	0.456	1.41	0.57	3.52
Object sucking or biting	**0.014**	**3.34**	**1.27**	**8.74**
At least one parafunctional oral habit	0.142	1.68	0.84	3.36
Fair- poor oral hygiene	0.119	1.78	0.86	3.69
Moderate- severe gingivitis	**0.048**	**2.13**	**1.01**	**4.50**
Moderate- extensive caries	**0.035**	**4.54**	**1.11**	**18.54**
Presence of DMFT	**0.031**	**2.16**	**1.07**	**4.36**
Presence of DMFS	**0.031**	**2.16**	**1.07**	**4.36**

*D* Decay, *M* Missing, *F* Filling, *T* Teeth, *S* Surface

There was a weak correlation between PMUM-SF score and plaque index (r = 0.17, *P* = 0.027), gingival index (*r* = 0.23, *P* = 0.004), max ICDAS score (*r* = 0.17, *P* = 0.034), DMFT (r = 0.19, *P* = 0.019) and DMFS (*r* = 0.19, *P* = 0.015) scores whereas there was no correlation between PMUM-SF score and dmft (*r* = -0.05, *P* = 0.474) and dmfs (*r* = -0.06, *P* = 0.420) scores. A moderate-high PMU increased the risk for moderate-severe gingivitis, moderate-extensive caries, presence of DMFT and presence of DMFS [Odds (95% CI) = 2.13 (1.01–4.50), *P* = 0.048; 4.54 (1.11–18.54), *P* = 0.035 and 2.16 (1.07–4.36), *P* = 0.031, respectively]. Moderate-high PMU was not associated with poor oral hygiene (*P* > 0.05) (Table 3.

## Discussion

To the best of our knowledge, this is the first study to investigate the relationship between oral habits, oral health and the PMU in school-age children. In this study, 57.5% of the children had at least one oral parafunctional habit, and moderate-high PMU increased the risk for object sucking/biting habit. Besides, 54.9% had daily toothbrushing habits, and moderate-high PMU decreased the probability of having a daily toothbrushing habit. According to the multivariate analysis result, moderate-high PMU was found to be associated with poor gingival status and more severe dental caries.

The PMUM-SF scale predicts PMU—screen media addiction in children independently from gender, duration of screen-time and type of media device [11]. Differently from these previous studies, we investigated PMU in our study. PMU is an addictive pattern of engagement with a variety of different screen activities (internet use, social media use, video gaming or mobile phone use) in a dependent, problematic manner [26]. The measure of PMU reveals the child's overall functioning above total daily screen time and type of used media. Originally, the present study revealed a significant relationship between PMU and oral health. We showed that a moderate-high PMU was related with reduced daily toothbrushing and reduced age-appropriate toothpaste use as well as increased gingival inflammation and presence of both caries and activity compared with low PMU. However, previous studies investigating the relationship between media use and oral health commonly had focused on the screen time or type of media used by children or adolescents. The study of Tsuchiya et al. [6] indicated a type-specific unfavorable impact of screen viewing on oral health behavior in the children aged 6–15 years. The authors have found an association between excessive video game playing (> 2 h/d), however, not with TV viewing, and also lower daily toothbrushing frequency (< 2 times/day). Besides, excessive video game playing was associated with unhealthy dental behavior which is defined as a lower brushing frequency regardless of the awareness of dental caries. Doitchinova et al. [27] have reported a correlation between prolonged TV viewing and dental caries activity in the children aged 6–12 years. In adolescents, problematic internet use -the excessive and disruptive nonessential use of the Internet- was associated with a low frequency of toothbrushing, gingival bleeding, tooth pain and neglect of dental checkups [27, 28]. In late adolescents, excessive computer use (> 3 h/d) was associated with oral hygiene neglect, absence from school due to oral pain, bleeding after probing the gingival pocket, less healthy periodontium, and decayed teeth [29]. Another study conducted on late adolescents demonstrated that problematic internet use was associated with negative oral health practice and gingivitis [30].

In the present study, 73.2% of the children did not follow the recommendation of twice-daily toothbrushing while 87.6% did not follow the recommendation of dental checkup every 6–12 months and only 12 (7.8%) had no PMU. The percentage of Australian children not following the recommendation of twice-daily toothbrushing and a regular dental checkup once every 6–12 months was found to be 66.9% and 62.9%, respectively. Also, more than two-thirds of Australian children did not adhere to recommended screen-time while more than half of parents were interested in receiving information about good oral health and screen-time practices [31, 32]. In line with these results, we can suggest that parental awareness about protective oral health and media use practices should be expanded in the Turkish population. For this purpose, dental visits may involve instructing parents about recommended preventive child health behaviors. In addition, instructive brochures about oral hygiene and healthy digital habits can be prepared to exhibit on school boards since problematic internet use was found to be negatively associated with toothbrushing after lunch at school [33].

In the present study, the children with low PMU were more likely to experience good oral habits leading to good oral hygiene. However, complying with recommendations on screen time, internet use, and dental practices have been reported to be less probable in the boys and children/adolescents from low socioeconomic backgrounds and urban residences [27, 33]. On the contrary, a previous study, similarly with our study, reported no significant difference between children with and without problematic internet use in terms of gender and socio-demographic characteristics [34]. Consistently with our findings, previous evidence manifested an association between problematic internet use and poor self-care and poor oral hygiene [33–35]. As it might be expected, PMU habits may lead to difficulties in performing daily routines.

There was no significant association between moderate-high PMU and following regular dental checkups in this study. However, a negative association was reported between heavy internet use and receiving an annual dental checkup in collegians [36]. Time and attention paid for regular dental visits may have prevented addictive media use behaviors in young population. Since childhood dental visits were under parental control, parents may have protected their children from missing regular dental visits.

Our findings indicate that PMU may deteriorate oral health even if the child spends the screen-time while using media as recommended. On the other hand, among adolescents, internet and social media used to gain information on oral health has been reported not to increase the prevalence of caries and the number of untreated caries [37]. Furthermore, healthier digital habits and rational media use should be encouraged in children and adolescents.

Our study expands the literature on experience of the children regarding oral health problems by demonstrating that moderate-high PMU may be linked to oral parafunctional habits. Park et al. [33] have revealed a negative association between problematic internet use and self-reported oral disease symptoms in adolescents. These symptoms included tooth, tongue, gum, and cheek disorders as well as bad breath. A previous study reported a positive correlation between increased screen media use and increased oral parafunctional habits, and it was emphasized that undesirable habits such as compulsive use of screens may deteriorate psychological and physiological health and may develop or aggravate oral parafunctional habits [38]. Oral parafunctional habits are common during childhood and they are one of the important etiological factors that may lead to malformations in dentofacial structures. There may be multiple reasons of these adverse oral habits [39]. The present study notes that PMU may be one of the factors associated with oral parafunctional habits. Although we detected a weak correlation, we can suggest that the number of oral parafunctional habits may also increase as PMU increases.

The most commonly used screen media among children was television in low PMU group while smart phone the most common media type in moderate-high PMU group. Besides, use of tablet and computer was more common in moderate-high PMU group. A recent study with a particular emphasis on screen media addiction among Turkish adolescents reported that the most common intentional uses with the digital devices were encountered towards social media and communication [40]. Consistently, sample profile of our study exhibiting screen-based media device use manifested that use of mobile and web-enabled device may be relevant to PMU.

The originality of this study is measurement of PMU which is an addictive behavior in children. In this study, statistical power was %99 in comparing the means of DMFT scores of low- and moderate-high PMU groups (OpenEpi calculator, http://www.openepi.com/Power/PowerMean.htm). The strength of our study is its emphasizing the associations of PMU with oral parafunctional habits, adverse oral health behaviors and poor oral health. Since we have identified an association between moderate-to-high PMU and the presence of gingivitis and dental caries, we can suggest that providing key recommendations about children’s health can be considered as a component of the dental visits. Receiving information from pediatric dentists not only about oral health, but also about media use, may help parents to establish healthy lifestyle habits in their children.

There are several limitations of this study. First, the cross-sectional design is not appropriate to determine the temporal relationship of PMU with oral habits and oral health status. Second, we obtained no information on dietary behavior, particularly the consumption of sugary foods or beverages during media-use sessions that could influence the association of PMU with parafunctional oral habits and poor oral health. However, since all children participating in this study used screens for less than 2 h, we can assume that they consumed a limited amount of cariogenic food and beverage during screen use. In addition, the normal weight of the children participants of the study indicating no underweight, overweight or obesity may alleviate this limitation of the study. Finally, we obtain no information about stress or parental smoking. We have evaluated sleep duration, however, we did not take sleep quality into consideration. However, both inadequate sleep duration and poor sleep quality have been associated with dental caries and oral symptoms [41, 42]. Thus, it is needed to carry out further prospective studies based on additional comprehensive variables. It is recommended to conduct a future research with a large representative sample of Turkish school children to confirm the results of the this pilot study.

## Conclusions

Problematic media use is an entity over excessive screen-time while some oral parafunctional habits and poor oral health may be related to PMU in children. Hence, it is needed to carry out an oral health screening in Turkish children with PMU and to educate their parents on this matter. It is essential to inculcate good oral habits and behaviors in the children by emphasizing the effects of PMU on oral health.

## Data Availability

The data that support the fndings of this study are available from the corresponding author upon reasonable request.

## Abbreviations

PMU: Problematic media use
PMUM-SF: Problematic Media Use Measure Short Form
ICDAS-II: The International Caries Detection and Evaluation System
DMFT/S (for permanent dentition), dmft/s (for primary dentition): Decayed (D, d), missing (M, m), and filled (F, f) tooth (T/t) /surfaces (S/s)
CI: Confidence Interval
IQR: Interquartile Range
ORs: Ods ratios
ADA: The American Dental Association
WHO: World Health Organization
